# Letter to the Editor regarding the Article “Effects of Intra-articular Bone Marrow Aspirate Infiltration in the Treatment of Knee Osteoarthritis: A Clinical Study Comparing BMA versus Corticosteroid and Genicular Block.” *Rev Bras Ortop* 2025;60(2):s00451809512. DOI:10.1055/s-0045-1809512

**DOI:** 10.1055/s-0046-1817129

**Published:** 2026-04-28

**Authors:** Rodrigo Monari, Carlos Henrique Pfiffer, Amadeu Davoglio Lorga, Morghana Fin

**Affiliations:** 1 Hospital Santo Antônio, Blumenau, SC, Brazil; 2 Hospital Unimed, Blumenau, SC, Brazil; 3 Clínica Monari, Blumenau, SC, Brazil

Dear Editor-in-Chief of the *Brazilian Journal of Orthopedics and Traumatology* , Dr. Geraldo Motta,

After carefully reading the article by Clazzer et al., 1 we consider it pertinent to make some comments regarding the design and interpretation of the results presented.

1. Methodological Aspects
  This study, although prospective and randomized, has significant limitations. Fifteen patients were lost to follow-up, resulting in only 35 subjects evaluated over 6 months. This reduction compromises the originally planned statistical power. Furthermore, there is no detailed sample size calculation for relevant clinical outcomes, nor are the randomization criteria clearly described.
2. Conceptual Confusion about the Intervention
The authors describe the intervention group as being treated with bone marrow aspirate (BMA). However, according to their own methodology, the BMA was mixed with dexamethasone and injected intra-articularly. This point is critical because the literature indicates that glucocorticoids negatively affect mesenchymal stem cells, decreasing their viability, proliferation, and chondrogenic differentiation. 2 Such conduct contradicts the principles of regenerative medicine and makes it difficult to attribute the outcomes to BMA alone.
3. Interpretation of Results
  Clinical findings showed a significant difference only in the pain domain of the Western Ontario and McMaster Universities Arthritis Index (WOMAC) after 6 months, with no significant improvement in stiffness or function. Even so, the authors conclude that BMA represents a viable therapeutic option. We consider this statement premature, as it relies on limited outcomes and short follow-up. Unfortunately, we have noted that the Instagram page of the Brazilian Society of Orthopedics and Traumatology mistakenly claims that the study resulted in an improvement in pain and function.
